# An Illustrated Scoping Review of the Magnetic Resonance Imaging Characteristics of Canine and Feline Brain Tumors

**DOI:** 10.3390/ani14071044

**Published:** 2024-03-29

**Authors:** James L. May, Josefa Garcia-Mora, Michael Edwards, John H. Rossmeisl

**Affiliations:** 1 Veterinary and Comparative Neuro-Oncology Laboratory, Department of Small Animal Clinical Sciences, Virginia-Maryland College of Veterinary Medicine, Virginia Tech, Blacksburg, VA 24061, USA; jameslevimay@vt.edu (J.L.M.); jkgarciam@vt.edu (J.G.-M.); 2 Department of Small Animal Clinical Sciences, Virginia-Maryland College of Veterinary Medicine, Virginia Tech, Blacksburg, VA 24061, USA; mredward@vt.edu

**Keywords:** cat, choroid plexus tumor, companion animals, diffusion imaging, dog, glioma, magnetic resonance, meningioma, neuroimaging, perfusion imaging, spectroscopy

## Abstract

**Simple Summary:**

Magnetic resonance imaging is an invaluable tool for the diagnosis of brain diseases in veterinary medicine. In this review, we provide a comprehensive summary of the magnetic resonance imaging appearances of and diagnostic approach to brain tumors affecting dogs and cats. Characteristic imaging features often allow for accurate prediction of commonly encountered types of brain tumors in clinical practice, such as meningiomas, gliomas, and pituitary tumors. However, a limitation of magnetic resonance imaging remains that some types of brain tumors, as well as other causes of brain disease, display similar imaging features, which can lead to diagnostic uncertainty and errors. There are also several uncommon to rare variants of brain tumors for which published magnetic resonance imaging descriptions are extremely limited or non-existent, especially in cats. The recent development and clinical usage of quantitative imaging and artificial intelligence techniques show promise for improving the ability of magnetic resonance imaging to correctly diagnose brain tumors and discriminate different tumor types.

**Abstract:**

Magnetic resonance imaging (MRI) is used pervasively in veterinary practice for the antemortem diagnosis of intracranial tumors. Here, we provide an illustrated summary of the published MRI features of primary and secondary intracranial tumors of dogs and cats, following PRISMA scoping review guidelines. The PubMed and Web of Science databases were searched for relevant records, and input from stakeholders was solicited to select data for extraction. Sixty-seven studies of moderate to low-level evidence quality describing the MRI features of pathologically confirmed canine and feline brain tumors met inclusion criteria. Considerable variability in data inclusion and reporting, as well as low case numbers, prohibited comparative data analyses. Available data support a holistic MRI approach incorporating lesion number, location within the brain, shape, intrinsic signal appearances on multiparametric sequences, patterns of contrast enhancement, and associated secondary changes in the brain to prioritize differential imaging diagnoses, and often allows for accurate presumptive diagnosis of common intracranial tumors. Quantitative MRI techniques show promise for improving discrimination of neoplastic from non-neoplastic brain lesions, as well as differentiating brain tumor types and grades, but sample size limitations will likely remain a significant practical obstacle to the design of robustly powered radiomic studies. For many brain tumor variants, particularly in cats, there remains a need for standardized studies that correlate clinicopathologic and neuroimaging data.

## 1. Introduction

Brain tumors are a common and significant cause of neurological dysfunction and death in dogs and cats [1,2,3,4]. Definitive diagnosis of intracranial neoplasia requires histopathologic examination of representative tissue. However, antemortem biopsy of brain tumors is infrequently performed in veterinary clinical practice due to the relatively limited availability of institutions with the experience and equipment required to perform stereotactic biopsy techniques, the potential for serious adverse events associated with biopsy, as well as the additional financial burden the procedure places on animal owners [1,5]. Given the practical constraints associated with brain biopsy in veterinary medicine, magnetic resonance imaging (MRI) of the brain is an indispensable tool for the presumptive clinical diagnosis of brain tumors or other possible etiologies of the observed neurological signs [1,6,7,8,9].

Since the incorporation of MRI into veterinary medical practice over 30 years ago, a body of literature has been generated regarding the MRI features of primary and secondary brain tumors that affect dogs and cats. The specificity of MRI has been demonstrated to exceed 90% for canine and feline brain neoplasms, although reported sensitivities for the classification of specific tumor types based on MRI features are highly variable [7,10,11]. While several reviews describing the MRI characteristics of canine and feline brain tumors have been published, additional studies expanding upon existing neuroimaging knowledge and introducing new MRI features of commonly encountered brain tumors of dogs and cats have emerged in parallel with the evolution and more widespread use of MRI, quantitative imaging techniques, and computational analytical methods [9,12,13,14].

Given the heterogeneity of both the types of tumors and different species represented in this topic, here we provide a scoping review of the MRI characteristics of naturally occurring brain tumors affecting dogs and cats [15]. The objectives of the review are to: (1) provide a contemporary summary of the qualitative and quantitative MRI features of primary and secondary brain neoplasms of dogs and cats; (2) develop an imaging feature-based conceptual framework to facilitate the generation of neuroimaging differential diagnoses in clinical practice; and (3) to identify existing knowledge gaps in the neuroimaging of intracranial tumors in companion animal medicine. We further provide representative MRI illustrations of pathologically confirmed canine and feline brain tumors using cases derived from our veterinary neuro-oncology practice.

## 2. Materials and Methods

The study was conducted in accordance with Preferred Reporting Items for Systematic reviews and Meta-Analyses extension for Scoping Reviews (PRISMA-ScR) guidelines [16]. To derive and summarize the MRI features of canine and feline intracranial tumors, the PubMed and Web of Science databases were searched for articles published from January 1995 through December 2023 using the medical subject headings (MeSH) terms: animal/canine/dog, brain mass, brain metastases, brain neoplasia, brain tumor(s), magnetic resonance imaging, veterinary; animal/feline/cat, brain mass, brain metastases, brain neoplasia, brain mass, brain tumor(s), magnetic resonance imaging, veterinary; and dog/cat, choroid plexus tumor, glioma, histiocytic sarcoma, lymphoma, melanoma, meningioma, pituitary tumor. The dates included in the database searches were chosen as representative of the time period during which specialty veterinary practices in the developed world began to install and utilize MRI as part of the routine clinical evaluation of dogs and cats with intracranial disease [17]. The most recent database search was performed on 4 January 2024. The references of several other broadly scoped veterinary MRI review articles by Boss [18], Greco [19], Hecht [20], LeBlanc [21], and Vite [22] were also examined for potential data sources. The search was supplemented by entering the digital object identifier for each record returned during the initial database queries into the ‘citationchaser’ freeware tool and performing forward and backward citation chasing [23]. Studies identified in the composite search were imported into a bibliographic management program (EndNote v 20.6, Clarivate, London, UK), and duplicate records were removed using library management tools. For inclusion in the review, records were required to have been published in English and contain at least 3 cases with MRI descriptions of cytologically or histopathologically confirmed, naturally occurring intracranial neoplasms. Records describing primary head, neck, or skull cancers with local invasion into the brain were excluded.

To select the MRI tumor features for data extraction, we first engaged a global network of stakeholders that routinely utilize MRI in veterinary practice, including neurologists, radiologists, and radiation oncologists, using a web-based survey designed to identify MRI sequences and practices utilized in the clinical management of dogs and cats with brain disease [24]. MRI sequences and techniques that were used by ≥50% of survey respondents were then cross-referenced to a subset of published veterinary brain tumor studies to identify common denominator variables for possible data extraction [1,2,6,7,8,9,10,11,12,13,14,25,26]. A 10-member panel consisting of internal institutional stakeholders and study investigators (two neurologists, two neuroscientists, one radiologist, one oncologist, one neurology resident, one cancer biologist, one bioinformaticist, and one neuropathologist) then selected the final MRI data to be mined by consensus from these common denominators.

For each tumor type (and/or tumor grade) in each species, the following MRI data were extracted: lesion number (solitary or multifocal/diffuse); lesion location (extra-axial, intra-axial, intraventricular, sellar/parasellar, or mixed/multiple locations); lesion shape; lesion margination (well or poorly defined); lesion signal intensity on T1-weighted sequences pre- (T1W) and post-intravenous administration of gadolinium (T1W + Gd) based contrast agents; lesion signal intensity on T2-weighted (T2W), T2W-fluid attenuated inversion recovery (FLAIR) and/or proton density (PDW) sequences; lesion signal intensity on diffusion weighted image (DWI) sequences and apparent diffusion coefficient (ADC) maps; lesion signal intensity on gradient recalled echo T2*-weighted (T2*) or susceptibility weighted (SWI) sequences; the presence/absence and severity of contrast-enhancement (mild, moderate, marked); the pattern of contrast enhancement (uniform/homogeneous, partial/heterogeneous, ring); and associations with locoregional effects in the brain (peritumoral edema, mass effect, meningeal lesions, brain herniations, calvarial lesions). All signal intensities are reported relative to normal-appearing gray matter. Data extraction was independently performed by two investigators.

The level of evidence provided by each study included was critically appraised using the hierarchal system developed by the Oxford Centre for Evidence-Based Medicine, with the exception of those studies that reported radiomic, machine learning, and/or artificial intelligence-derived quantitative data (collectively referred to as radiomic studies) [27]. The quality of radiomic studies was evaluated with the Quality Assessment of Diagnostic Accuracy Studies (QUADAS-2) criteria, as adapted and recommended for radiomics [28]. Records meeting inclusion criteria consisted of those of moderate to low-level evidence quality, with considerable variability in data collection and reporting or high risks of bias due to limited sample numbers [27,28]. Since this precluded homogeneous and robust comparisons of results, we made no attempt to statistically analyze data included in the scoping review. As a result, extracted data are reported as summary text or tables.

To generate representative MRI of the various tumors reviewed and provide visual reinforcement of the narrative and tabular review data, the central nervous system tissue biorepository of the Veterinary and Comparative Neuro-oncology Laboratory was searched using the same MeSH terms used for the scoping review data sources to identify cases of dogs and cats with histologically confirmed brain tumors and diagnostic brain MRI studies available for review on the institutional picture archiving and communication system. All MRI illustrations appearing in this review are projected using common veterinary imaging conventions, with the left side of the animal’s head appearing on the reader’s right in transverse and dorsal planar images and rostral appearing at the top of dorsal planar and to the left of sagittal images [12]. In addition, each row of image panels in each figure represents data obtained from the same animal (case).

## 3. Results

**Figure 1:**
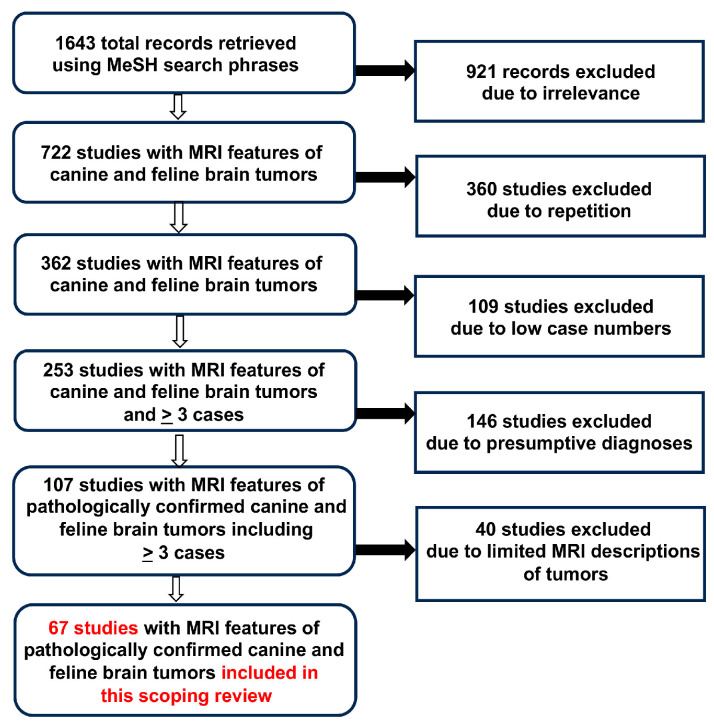
Flowchart for inclusion of search records in scoping review.

After the exclusion of studies describing experimentally induced brain tumors and other irrelevant records, de-duplication of records appearing repeatedly in our database searches using the different MeSH search phrases, and removal of studies that contained <3 cases, unconfirmed diagnoses, or limited MRI data, we identified 67 articles (Figure 1) that fulfilled the inclusion criteria, with these studies reporting a total of 1630 canine (from 59 studies) and 125 feline cases (from 9 studies) with tumors [2,6,7,8,9,10,11,12,13,25,26,29,30,31,32,33,34,35,36,37,38,39,40,41,42,43,44,45,46,47,48,49,50,51,52,53,54,55,56,57,58,59,60,61,62,63,64,65,66,67,68,69,70,71,72,73,74,75,76,77,78,79,80,81,82,83]. A total of 62/67 studies were classified using Oxford hierarchal evidentiary criteria [27]: 45/62 (73%) were level 2b retrospective cohort studies [2,6,7,8,9,10,11,25,26,29,31,32,33,35,36,37,40,41,42,48,51,52,53,54,55,56,57,58,59,63,64,65,66,67,69,70,71,72,74,77,78,81,82,83], 14/62 (23%) were level 4 case series each describing ≤10 animals [30,38,43,47,49,50,58,61,62,73,75,76,79,80], and 3/62 (4%) were level 3a ‘minus’ reviews containing source data quality heterogeneity [12,13,14]. Five radiomic studies were identified [34,44,45,46,68], with all radiomic studies having QUADAS-2 scores ≤6, and thus were considered to provide low-level evidence [28]. The radiomic studies included had a high risk of bias within the patient selection domain, manifesting as small sample sizes given the number of variables tested or imbalances among analytical subgroups [28].

**Figure 2:**
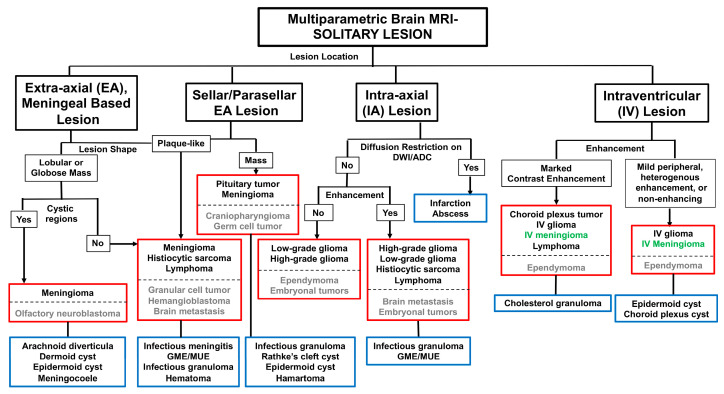
MRI feature-based differential diagnostic algorithm for solitary intracranial lesions in dogs and cats. Neoplastic diagnostic considerations based on imaging features are included in red boxes; non-neoplastic diagnoses are in blue boxes. Common tumor types are indicated in black text; uncommon to rarely encountered tumors in gray text. Tumors are predominantly found in cats, indicated in green text.

For the generation of a prioritized list of differential diagnoses based on the MRI characteristics of brain lesions, a recurrent theme that emerged from the literature was to identify the number of lesions (solitary; multifocal/diffuse) present and then classify the neuroanatomic location of the pathology. Four predominant patterns of neuroanatomic distribution were identified, which included extra-axial, meningeal-based, extra-axial sellar/parasellar, intra-axial, or intraventricular lesions, with subsequent decision-making within each neuroanatomic location driven by a variety of secondary lesion features such as lesion shape, signal characteristics, or contrast-enhancement patterns [2,6,7,8,9,10,11,12,13,14]. This approach has been contextualized for solitary canine and feline brain tumors in Figure 2. This algorithm is intended to provide guidelines to assist with the refinement of potential diagnoses based purely on MRI data. However, it is paramount that an evaluation of each patient that includes historical, physical examination, and clinicopathologic data, as well as diagnostic imaging findings, is used to create a relevant and comprehensive list of differential diagnoses for each case [1].

### 3.1. Solitary Extra-Axial, Meningeal Based Lesions

This extra-axial classification scheme refers to lesions arising from the meninges, interior to the calvarium, and exterior to the neural parenchyma [10,12,13,14] but intentionally excludes extra-axial masses arising in sellar/parasellar or intraventricular locations (Figure 2) for the purposes of refining differential diagnostic considerations. Meningiomas are the most common extra-axial, meningeal-based tumor in both dogs and cats, with other sporadic neoplastic differential diagnostic considerations including histiocytic sarcoma (HS), lymphoma, solitary brain metastases, granular cell tumor (GCT) mesenchymal non-meningothelial tumors (hemangioblastoma), and embryonal tumors (olfactory neuroblastoma) [2,4,9,12,14,61,62].

Imaging features that are shared among extra-axial, meningeal-based lesions include a broad base of dural contact, an absence of normal brain parenchyma present between the lesion and its boundary of dural or dural-calvarial contact, and expansion of the subarachnoid space [2,7,9,10,11,12,13,14]. Additionally, as the pachymeninges are not protected by the blood–brain barrier, contrast-enhancement is a common denominator among neoplastic and non-neoplastic extra-axial, meningeal-based lesions [2,7,10,12,14,31,32]. This contrast enhancement frequently also includes a ‘dural tail sign’, which is a plaque-like to the linear region of thickened and enhancing dura mater present on T1W + Gd images that extend away from the epicenter of an extra-axial, meningeal-based lesion [12,48,51]. Although the dural tail sign occurs more frequently in association with neoplasms, it is not a specific feature of brain tumors, having also been observed in association with inflammatory diseases [7,14].

#### 3.1.1. Meningioma

**Figure 3:**
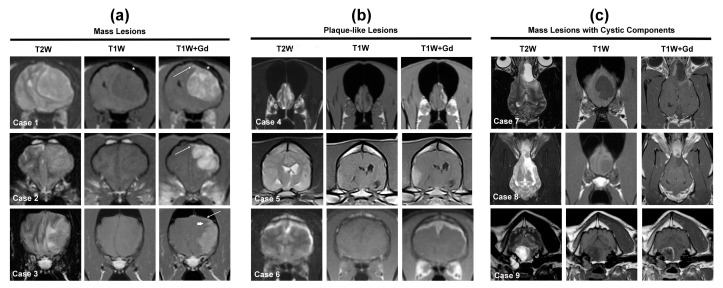
MRI features of solitary, extra-axial, meningeal-based neoplastic lesions in dogs and cats. Cases 1–3 (**a**) illustrate mass lesions, Cases 4–6 (**b**) are plaque-like lesions, and Cases 7–9 (**c**) are mass lesions with significant cystic components. Case 1—feline Grade I cerebral convexity meningioma. The tumor is broad-based and well-demarcated, associated with mass effect manifested as a falx shift and compression of the left lateral ventricle, and is T2W iso- to-hyperintense, T1W hypointense, and markedly and heterogeneously contrast enhancing. Note the dural tail sign (arrow) and calvarial hyperostosis (*). Case 2—canine Grade II cerebral convexity meningioma with similar signal appearances as in Case 1, with dural tail sign (arrow). Case 3—canine intracranial histiocytic sarcoma. The tumor is T2W iso- to hyperintense, T1W iso- to hypointense, uniformly contrast enhancing, and has a dural tail feature (arrow) with an extension of contrast enhancement into the leptomeninges of the sulci (arrowhead) and associated mass effect and perilesional T2W hyperintensity representing edema. Case 4—canine Grade I plaque-like cerebral convexity meningioma. Case 5—canine granular cell tumor appears as a plaque-like lesion that is iso-to hyperintense on T2W images, T1W hyperintense, markedly contrast enhancing, and associated with mass effect. Case 6—feline intracranial B-cell lymphoma manifesting as marked T2W hyperintense, T1W hypointense, markedly contrast-enhancing, and plaque-like thickening of the pachymeninges of both cerebral hemispheres. Case 7—canine Grade I cystic olfactory meningioma. The mass is well demarcated, with cystic regions represented by areas of marked and uniform T2W hyperintensity and T1W hypointensity. Solid portions of the tumor are contrast-enhancing and surround the cystic region. Perilesional T2W hyperintensity represents edema in the caudal aspect of the mass. Case 8—canine olfactory neuroblastoma, which shares imaging features with cystic meningiomas. The mass is heterogeneously T2W hyperintense, with the caudal cystic aspect of the mass being markedly hyperintense, T1W iso- to hypointense, and the rostral portion of the tumor demonstrates marked but heterogenous contrast enhancement. Note the solid, enhancing portion of the mass extending from the nasal cavity through the cribriform plate into the calvarium and the extensive perilesional T2W hyperintensity (edema) extending caudally along the subcortical white matter tracts. Case 9—canine Grade I microcystic meningioma of the cerebellopontine angle. Intratumoral cysts account for the T1W and T2W signal heterogeneity, and the mass demonstrates peripheral ring-type enhancement. Note the presence of fluid material in the middle ear as well as the marked neurogenic atrophy of the muscles of mastication ipsilateral to the tumor.

Meningiomas account for 45% and 58% of all primary brain tumors in dogs and cats, respectively, and are the most frequently diagnosed extra-axial, meningeal-based tumors in both species [2,3,9,10,31,32]. Of the 125 feline brain tumor MRI cases included in this review, 71/125 (57%) were meningiomas. Meningiomas are histologically graded using World Health Organization criteria, and the vast majority of feline meningiomas are Grade I tumors [12,13]. In dogs, Grade I and II (atypical) meningiomas each occur with considerable frequency, while Grade III (malignant) variants are rare [48]. Meningiomas may be further described according to their anatomic site in the brain as basilar, cerebellopontine angle (CPA), cerebral convexity, cerebellar convexity, falcine, foramen magnum, olfactory, parasellar, parasagittal, or tentorial (Figure 3). In the dog, 66–85% of meningiomas occur in rostrotentorial locations, with olfactory, cerebral convexity and parasagittal being the most frequently reported sites of occurrence [2,10,29,30,31,32,40,48]. In the cat, ~90% of meningiomas are rostrotentorial, with tumors located over the cerebral convexities accounting for nearly 50% of all feline meningiomas [3,9]. Cats also have a propensity to develop third ventricular meningiomas, which develop from the tela choroidea [3]. This specific tumor location is included with intraventricular masses in this review. Approximately 70–80% of canine and feline meningiomas manifest as spherical, ovoid, or lobular-shaped masses (Figure 3), with the remaining tumors assuming a plaque-like morphology [9,48]. Although most meningiomas manifest as solitary masses, up to 17% of cats with meningiomas diagnosed by MRI had multiple, synchronous, and discrete meningiomas [3,49]. Multiple meningiomas are infrequently reported in dogs [7,48].

The reported MRI signal intensities of canine and feline meningiomas are variable (Table 1) and not specific for any particular extra-axial meningeal-based tumor or non-neoplastic lesion [7,11]. The majority of canine and feline meningiomas are isointense to hypointense on T1W images and heterogeneously hyperintense on T2W/FLAIR images [9,10,30,31,32,48,49]. Although contrast-enhancement occurs nearly universally in canine and feline meningiomas (Table 1), studies report considerable variability as to whether contrast-enhancement occurs homo- or heterogeneously (Table 1), as well as a wide range of the incidence of the dural tail sign in canine meningiomas [9,10,30,31,32,39,48,49]. The majority of meningiomas display well-demarcated borders, particularly on T1W+Gd images (Figure 3, Cases 1, 2, 4, 7, 8), and a sharply defined border has been shown to be predictive for the presence of a neoplastic rather than inflammatory or vascular lesions on MRI studies [7].

Meningiomas are also the primary diagnostic consideration for extra-axial meningeal-based lesions that contain large solitary or multifocal cysts (Figure 3, Cases 7–9), which appear as well-demarcated T1W hypointense, T2W hyperintense, non-enhancing spherical to multiloculated regions [47,48]. Cystic meningiomas have comprised 13–32% of canine meningiomas included in the studies in this review [2,7,10,30,31,32,40,48,52,53,54], although an additional subset of meningiomas also contain smaller foci of intratumoral cysts/fluid or intratumoral hemorrhage, with hemorrhage appearing as conspicuous signal voids or susceptibility artifacts on T2*GRE or SWI [9,14,48,54].

Meningiomas are frequently associated with secondary changes in the brain (Table 1), such as peritumoral edema and mass effect [2,9,41,48]. Peritumoral edema, which is T1W hypointense and T2W/FLAIR hyperintense, is predominantly vasogenic with a propensity to track along white matter tracts adjacent to the tumor. Peritumoral edema is often subjectively defined as mild, moderate, and severe, with most sources considering edema involving ≥3 cerebral lobes or an entire hemisphere to be severe [2,10,31,32]. Mass effect can manifest in many forms, including effacement of sulci, shifting of midline structures, compression of ventricles, or the presence of brain herniations [12,13,14,41]. Cats with meningiomas frequently have brain herniations on MRI, with a reported incidence of 63–100%, which is likely attributable to the relatively high tumor volume present in most cats at the time of diagnosis [9,41]. Brain herniations in dogs with meningiomas also occur commonly, although the incidence is highly variable, as this imaging feature of mass effect is not always specifically reported [41]. Canine meningiomas have also been shown to cause displacement of local blood vessels in three dimensional time-of-flight magnetic resonance angiographic (3D TOF MRA) studies [53]. Meningiomas are also the most likely extra-axial, meningeal-based tumors to cause secondary changes in the skull adjacent to the tumor [9,40,52]. Bony changes most often consist of thickening and sclerosis of the calvarium (calvarial hyperostosis) but can also rarely include lysis [2,9,40,52]. Calvarial hyperostosis (Table 1; Figure 3, Case 1) is observed much more frequently in feline meningiomas [9,40,52].

There are currently no qualitative MRI imaging criteria that reliably allow for the prediction of the histological grades of meningioma [48]. The use of quantitative imaging techniques, such as derivation of the ADC from DWI or diffusion tensor imaging (DTI) sequences, provides data about the diffusion properties of water in tissue [26,35,37]. Additionally, fractional anisotropy (FA) may be calculated from DTI, with the FA providing data about the magnitude of directional diffusion of water, which is considered an indirect index of the microstructural integrity or properties of tissue [26,35]. In human neuro-oncology, ADC values have been shown to correlate with tumor cellularity, tumor grade, and prognosis [84]. Reduction in the water diffusion is reflected by low ADC values and high DWI signal, such as in highly cellular or fibrous tumors, and high FA values correlate with tumor density [26,35]. One study in dogs demonstrated that ADC values of atypical and malignant meningiomas are significantly lower than those of Grade I tumors [42], and thus, ADC values may be helpful for the non-invasive grading of meningiomas (Supplementary Table S1). FA values have also been shown to correlate with biomechanical tissue properties such as stiffness, and it has been reported that compared to dogs, feline meningiomas have significantly lower ADC and higher FA values, which is consistent with intraoperative observations that the consistency of feline meningiomas tend to be firmer than those of dogs [26].

Several studies have reported on the accuracy of qualitative MRI features to diagnose meningiomas. Investigators correctly identified 24/25 (96%) feline meningiomas from a population of 45 cats with brain tumors but were aware that each MRI study contained at least one brain tumor [9]. In studies by Snyder et al. [2], Rodenas et al. [10], and Thomas et al. [32], MRI feature analysis resulted in the correct diagnosis of 8/9 (88%), 6/9 (67%) and 7/8 (88%) of canine meningiomas, respectively. In a study that included dogs with a broad range of neoplastic, inflammatory, and vascular brain diseases, MRI features were 59.6% sensitive for the detection of meningioma [11]. Mai et al. reported that the probabilities of experienced radiologists correctly identifying meningiomas from a population of dogs with extra-axial, meningeal-based tumors ranged from 79–94% [52].

#### 3.1.2. Histiocytic Sarcoma (HS)

HS represents a range of aggressive neoplastic entities, from which most arise from interstitial dendritic cells and which may occur as localized or disseminated diseases [52,55]. In the central nervous system (CNS), HS can occur as both a primary or secondary tumor [55]. Although CNS HS can have protean MRI manifestations, including solitary or multifocal/diffuse extra-axial masses, intra-axial masses, and mixed extra- and intra-axial lesions, we include HS in this section as a solitary extra-axial meningeal-based lesion is the predominant imaging presentation of HS in the canine brain [2,33,52,53,54,55,56]. In dogs, HS is the second most likely differential diagnosis for this type of mass appearance after meningioma [14,52,55]. Although CNS HS also occurs in cats, reports describing the MRI features of this tumor and meeting the inclusion criteria of this review were not identified.

There is considerable overlap in the MRI features between canine HS and meningiomas (Table 1). However, the presence of some features should increase the index of suspicion that HS is a more likely differential diagnosis than meningioma. Hypointensity of the lesion on T2W images is rarely observed in meningioma (Table 1) but is relatively common in HS [48,53]. On post-contrast T1W images, HS is also significantly more likely than meningioma to demonstrate contrast enhancement into the leptomeninges with invasion into and widening of the sulci (Figure 3, Case 3), and meningeal enhancement extending distant from the tumor bulk [52,53,54,55]. HS may also be more likely to present with severe peritumoral edema than meningioma [52,54], but the degree of edema associated with both these tumors can vary widely [53,54,55]. Compared to meningiomas, canine HS have significantly lower ADC values (Supplementary Table S1) and do not cause blood vessel displacement on 3D TOF MRA, although differences in FA values between meningiomas and HS were not found [53,54].

The accuracies of MRI features to diagnose HS in dogs have not been investigated nearly as extensively as meningiomas due to the infrequency with which definitively diagnosed CNS HS with correlative MRI studies have been reported [2,52]. The one HS in the report by Snyder et al. was incorrectly diagnosed [2], and Mai et al. reported that the probability of experienced radiologists correctly discriminating HS from meningiomas ranged from 76–92% [52].

#### 3.1.3. Lymphoma

The majority of lymphomas affecting the nervous system of dogs and cats secondarily involve the brain as part of a multicentric disease, with primary extranodal lymphomas accounting for 33% of feline and 4% of canine CNS lymphomas [2,3,4]. CNS lymphoma can also have multiple MRI manifestations, including solitary or multifocal/diffuse extra-axial masses, intra-axial masses, sellar or parasellar lesions, intraventricular lesions, and lesions involving multiple anatomic areas [2,9,10,11,12,57,58,59,60]. However, as with HS, lymphoma is included in this section, as focal extra-axial meningeal-based lesions are common imaging presentations (Figure 3, Case 6) of intracranial lymphoma in dogs and cats. In cats, lymphoma is the second most likely neoplastic differential diagnosis for this type of mass appearance after meningioma [9,57].

MRI signal characteristics of CNS lymphomas are non-specific, with the majority of lesions demonstrating hypo to iso-intensity on T1W images, hyper- to isointensity on T2W and PD images, and FLAIR iso- to hyperintensity [9,10,11,12,57,58,59,60]. Common features of CNS lymphomas are the presence of some degree of lesion contrast enhancement (usually moderate to marked in severity) irrespective of the anatomic location of the lesion(s), regional meningeal enhancement, ill-defined margins, and the presence of mass effect and peritumoral edema in cases presenting as solitary mass lesions [2,9,10,11,12,57,58,59,60]. The detection of extraneural lesions in the MRI scan field may increase the likelihood of a lesion being lymphoma, but this can also be observed in HS [55,57]. CNS lymphomas may also present with a dural tail sign [9,57,59]. Notably, brain MRI examinations may be normal in cases of CNS lymphoma [9].

Intravascular, or ‘angiocentric’, CNS lymphoma (IVL), which is characterized by intraluminal proliferation of neoplastic lymphocytes within arteries and veins, may present with characteristic MRI features. Cases of IVL affecting the brain typically feature multifocal T2W and FLAIR hyperintense and T1W iso- to hypointense intra-axial lesions that demonstrate variable contrast enhancement [60]. In addition, areas of restricted diffusion are present both in arterial and venous territories on DWI and ADC. On T2*GRE or SWI, tubular susceptibility artifacts, termed the ‘vessel susceptibility sign’, and additional intraparenchymal susceptibility artifacts are often noted [60]. These imaging findings reflect the presence of ischemic and hemorrhagic infarcts of variable chronicity caused by IVL-induced thrombi.

The use of MRI features to diagnose lymphoma has been the subject of very few reports, each describing very limited (e.g., 1–3) cases [2,9,10,11]. As a result, diagnostic sensitivities for lymphoma are highly variable, ranging from 0–100%, and should be interpreted cautiously.

#### 3.1.4. Other Rarely Encountered Extra-Axial, Meningeal Based Neoplasms

Here, we include non-meningothelial mesenchymal tumors (hemangioblastoma, primary intracranial sarcoma), embryonal tumors (neuroblastoma), and granular cell tumors (GCT), predominantly because each of these rare tumor variants may demonstrate MRI features that closely mimic the appearance of meningiomas [2,9,10,14,61,62]. Non-meningothelial mesenchymal tumors have also presented diagnostic challenges to neuropathologists, with some possibly having been previously classified as vascular hamartomas, undifferentiated sarcoma, or meningeal sarcomas [2,10,84]. Both hemangioblastomas and olfactory neuroblastomas (Figure 3, Case 8) may appear as extra-axial, markedly and heterogeneously contrast-enhancing masses involving the front-olfactory region and may contain hemorrhagic or cystic regions [9,14]. One feature of olfactory neuroblastoma that may help differentiate it from meningioma is the presence of cribiform plate lysis or extension of the mass from the nasal cavity through the cribiform into the cranial vault, although these cribiform changes may be minimal and can also rarely be seen with meningiomas [2,9,14]. GCT in dogs and cats are rare tumors of currently unknown histogenesis. These tumors also appear demarcated, plaque-like lesions involving the cerebral convexities, falx cerebri, or meninges of the skull base. In dogs, GCT may be iso- to hyperintense or hypointense on T2W images, with the majority demonstrating mild hyperintensity on pre-contrast T1W images (Figure 3, Case 5), strong homogenous contrast enhancement, significant mass effect, and perilesional edema [61,62].

### 3.2. Solitary Sellar and Parasellar Extra-Axial Mass Lesions

This group of lesions includes those contained within the sella turcica (sellar), which is the bony recess within the basisphenoid bone at the base of the skull, and those occurring dorsally, laterally, or ventrally adjacent to the sella (parasellar). The sella is normally occupied by the pituitary gland, which is composed of the adenohypophysis and neurohypophysis, with the neurohypophysis connected to the overlying hypothalamus by the stalk-like infundibulum [85,86]. Parasellar lesions may arise from numerous neurovascular structures, including the hypothalamus, ventral aspect of the third ventricle, regional cranial nerves (II, III, IV, ophthalmic branch of V, and VI), cavernous sinuses, or internal carotid artery [85].

#### 3.2.1. Sellar and Parasellar Mass Lesions of Pituitary Origin

**Figure 4:**
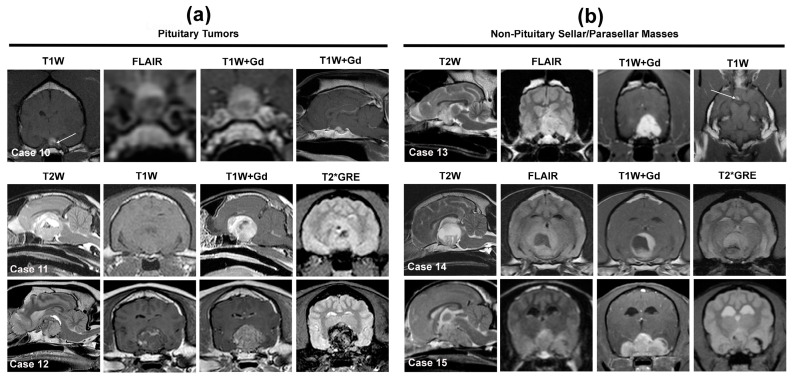
MRI features of solitary, extra-axial, sellar, and parasellar neoplastic lesions in dogs and cats. Cases 10–12 illustrate pituitary (sellar) tumors (**a**), and Cases 13–15 (**b**) non-pituitary origin tumors. Case 10—canine, cystic pituitary corticotroph adenoma. The contrast-enhancing, cystic adenohypophyseal mass displaces the T1W hyperintense neurohypophysis dorsally and the left of midline (arrow), such that it extends above the dorsum sella and contacts the hypothalamus. Case 11—feline pituitary somatotroph macroadenoma resulting in marked compression of the thalamus, third, and lateral ventricles. The tumor is T2W heterogeneously hyperintense, T1W iso- to hypointense, and markedly and heterogeneously contrast enhancing. Regions of intra-tumor hemorrhage appear as areas of T2W hypointensity corresponding with signal voids on the T2*GRE image. Case 12—canine pituitary corticotroph macroadenoma. The mass demonstrates a heterogeneous signal in all sequences due to regions of intratumoral hemorrhage, which appear hypointense on T2W and T2*GRE images and hyperintense on T1W images. Case 13—canine parasellar meningioma. The tumor is T2W and FLAIR heterogeneously hyperintense, demonstrates strong and uniform contrast enhancement, and extends into the pituitary fossa eccentrically, displacing the T1W hyperintense neurohypophysis away from the midline (arrow). Case 14—canine craniopharyngioma causing marked compression of the third ventricle and thalamus. The mass contains solid (T2W and FLAIR heterogeneously hyperintense and contrast enhancing) and cystic (uniformly T2W hyperintense and T1W/FLAIR hypointense, non-enhancing areas) components. Case 15—canine large B-cell lymphoma manifesting as markedly enhancing extra-axial mass involving the sellar and parasellar regions with compression and dorsal deviation of the thalamus and mesencephalon.

Pituitary tumors are the most common neoplastic sellar lesions. Pituitary tumors are the second most common secondary brain tumors in dogs and cats after hemangiosarcoma and lymphoma, respectively, and appear to almost exclusively arise from the adenohypophysis [2,3,12,86]. Corticotroph adenomas predominate in dogs and somatotroph adenomas (Figure 4) in cats [2,12,64,65,86]. Although there is some anatomic variability in the MRI appearance of pituitary glands, on T1W images, the normal gland typically appears as a T1W isointense and T2W isointense to hypointense peripheral rim of tissue, representing the adenohypophysis, with a central ovoid, triangular, or ‘V’-shaped focus of T1W hyperintensity, reflecting the vasopressin neurosecretory granules within the neurohypophysis [12,86]. The normal pituitary gland has a mildly convex shape; its dorsal margin should not extend above the rim of the dorsum sellae on sagittal images and typically is uniformly enhancing on post-contrast T1W images [86].

Pituitary tumors presenting in normal-sized pituitary glands (e.g., microadenomas) can be challenging to detect, especially in cases that do not display clinical signs of underlying endocrinopathy. Eccentric displacement of the T1W hyperintense neurohypophysis within the sella turcica or dorsally above the sella is a potential indicator of the presence of a pituitary mass (Figure 4, Case 10), although this may also be observed with incidental pituitary cystic lesions [12,86]. The use of dynamic contrast-enhanced MRI can facilitate the identification of small pituitary tumors via the quantification of kinetic changes in the enhancement of the adeno- and neurohypophysis [38]. The MRI diagnosis of large pituitary tumors, so-called macrotumors, is usually straightforward, although the determination of tumor functionality requires supporting clinical signs of endocrinopathy and hormonal testing [2,9,10,11,31]. These masses occupy the pituitary fossa, extend dorsally or laterally into the parasellar regions beyond the dorsum sellae, are T1W hypo- to isointense, T2W iso- to hyperintense, demonstrate marked contrast enhancement, and can be associated with mass effect and peritumoral edema [2,9,31,32,64,65]. The T1W, T2W, and post-contrast signal intensities are often heterogeneous (Figure 4, Cases 10 and 11) due to the presence of intratumoral cysts or hemorrhage [2,31,64,65]. Adenomas tend to be round, and invasive pituitary adenomas and adenocarcinomas are more likely to be larger and have irregular shapes and borders compared to adenomas, but the composite MRI features of adenomas and invasive or malignant pituitary tumors have enough overlap to preclude accurate prediction of histology [2,9,10,64,65].

#### 3.2.2. Sellar and Parasellar Mass Lesions of Non-Pituitary Origin

The predominant, non-pituitary origin neoplastic differential diagnoses for sellar and parasellar masses include meningioma (Figure 4, Case 13), lymphoma (Figure 4, Case 15), HS, 3rd ventricular choroid plexus tumors (CPT), cranial nerve sheath tumors, and hematogenous brain metastases, with the MRI features of most of these tumors included elsewhere in this review [2,12,48,55,57,58,59,63,81]. Other rare tumor types that should be considered for solitary masses in this location include ependymoma, GCT, craniopharyngioma (Figure 4, Case 14), optic nerve pathway or hypothalamic gliomas, and germ cell tumors [61,62,63,70].

### 3.3. Solitary Intra-Axial Mass Lesions

**Figure 5:**
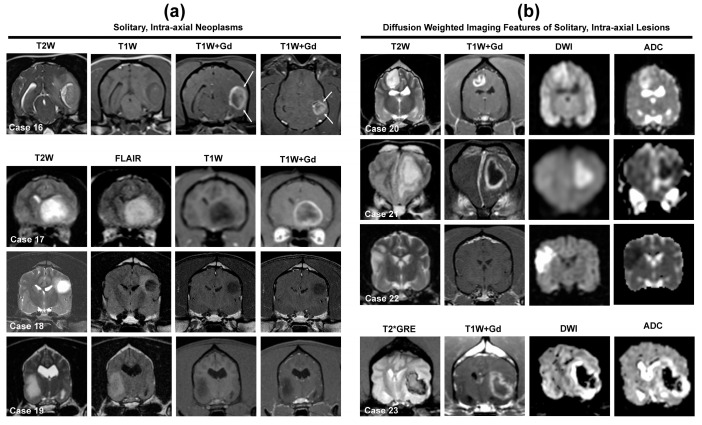
MRI features of solitary intra-axial gliomas of dogs and cats (**a**) and diffusion-weighted imaging appearances of solitary intra-axial lesions that can mimic neoplasms (**b**). Case 16—canine high-grade, ring-enhancing oligodendroglioma in the temporal and piriform lobes demonstrating the ‘claw-sign’ (arrows), mass effect, transtentorial herniation, and peritumoral edema. Case 17—feline diencephalic high-grade oligodendroglioma. The tumor is heterogeneously T2W/FLAIR hyperintense, T1W hypointense, and ring-enhancing. Case 18—canine low-grade, non-enhancing oligodendroglioma in the parietal lobe displaying the T2-FLAIR mismatch sign. Case 19—canine low-grade, T2W/FLAIR hyperintense, T1W hypointense, non-enhancing astrocytoma in the temporal piriform lobe. Case 20—canine high-grade, contrast-enhancing astrocytoma in the parietal lobe, which demonstrates non-uniform, ring-type enhancement and unrestricted diffusion, manifested as lesion hyperintensity on DWI and ADC images (T2W shine-through). Case 21—canine brain abscess with restricted diffusion (DWI hyperintense and ADC hypointense). Note the rim of T2W hypointensity surrounding the central T2W hyperintense lesion, uniform ring-enhancement of the abscess, and regional meningeal enhancement. Case 22—canine middle cerebral arterial ischemic infarction with restricted diffusion. The lesion is restricted to the cerebrocortical gray matter, is not associated with mass effect, and the parenchymal lesion is non-enhancing. Case 23—canine hemorrhagic brain infarction. The infarct is heterogeneously iso-to hypointense on T2*GRE, is ring-enhancing, associated with significant mass effect, and demonstrates T2W blackout (lesion hypointensity on DWI and ADC) due to susceptibility effects of hemorrhage. On DWI and ADC images, the peripheral hyperintensity surrounding the hypointense lesion core represents perilesional edema.

Intra-axial lesions arise from within the neural parenchyma [1,10]. On MRI, a key feature used to identify intra-axial lesions is the presence of brain tissue interposed between the lesion border and the meninges or meningeal-periosteal interface [14,67]. Difficulty correctly discriminating intra-axial from extra-axial brain lesions using MRI is commonly reported, particularly when the lesion is located peripherally [2,7,9,32,67,70]. The presence of a ‘claw-sign’ on MRI images (Figure 5, Case 16) occurs when an expansile lesion within an organ creates thinning of the surrounding parenchyma. In the brain, the claw-like appearance results from the formation of an acute angle at the border between the lesion and a pial surface, and the ‘claw-sign’ has been shown to further support, but not definitively represent, an intra-axial mass location [67].

The primary neoplastic differential diagnostic considerations for solitary intra-axial masses are neuroepithelial tumors, among which oligodendrogliomas and astrocytomas (i.e., gliomas) predominate in the dog (Figure 5, Cases 16–20), with other uncommon to rare possible differentials including undefined glioma (oligoastrocytoma), brain metastasis, ependymoma, lymphoma, HS, and embryonal tumors [2,9,10,31,32,55,66,67,68,69,70,71,72,73,74,75,76,77,78,79,80]. Gliomas can have wide-ranging MRI appearances, resulting in imaging features that may overlap substantially with brain abscesses, ischemic and hemorrhagic brain infarctions, fungal granulomas, immune-mediated encephalitides, leukoencephalopathies, and meningioma [2,6,7,8,10,12,13,14,67,77,78]. The routine inclusion of DWI or DTI sequences in multiparametric MRI imaging protocols is recommended to improve the ability to discriminate neoplastic from non-neoplastic intra-axial masses [6,7,8]. When generating a list of differential diagnoses for solitary intra-axial mass lesions, identifying the lesion T2W signal and diffusion pattern (non-restricted versus restricted) on DWI can facilitate this process (Figure 2 and Figure 5).

Neoplasms, granulomas, and meningoencephalitides are not usually associated with restricted diffusion and typically demonstrate lesion hyperintensity on T2W, DWI, and ADC images (Figure 5, Case 20), a DWI phenomenon termed T2 shine-through [6,8,35,37,66]. However, in the presence of vasogenic edema, these diseases may demonstrate T2 wash out, which appears as lesion hyperintensity on T2W and ADC images, but lesion isointensity on DWI. Restricted diffusion, characterized by lesion hyperintensity on T2W and DWI images corresponding to areas of hypointensity on ADC images, is a hallmark feature of pyogenic abscesses and brain infarctions (Figure 5, Cases 21 and 22) [6,8]. A T2 blackout pattern, which appears as lesion hypointensity in T2W, DWI, and ADC sequences, may be seen in hemorrhagic brain infarctions and tumors containing substantial intratumoral hemorrhage (Figure 5, Case 23).

#### 3.3.1. Gliomas (Astrocytoma, Oligodendroglioma, Undefined Glioma)

The inherent MRI features and signal intensities for gliomas are generally non-specific for neoplasia or for individual tumor type or grade [6,7,8,11,70,74,77,78,79,80]. The majority of gliomas have been documented in the forebrain, although gliomas of any type may occur anywhere in the brain parenchyma [2,70,74,77,78,79,80], and 60–89% of canine gliomas are high-grade tumors [66,69,70,77,78,80]. Gliomas, as well as other intra-axial neoplasms, are often T1W hypo- to isointense and T2W/FLAIR hyperintense (Figure 5a), but heteregeneous signal intensities are common [2,7,8,9,10,11,31,32,70,71,77,78].

Signal heterogeneity may arise from the presence of intratumoral cysts or hemorrhage, which occur in 30–80% and 25–50% of canine gliomas, respectively [2,10,66,69,70,74,77,78,79,80]. Some degree of mass effect and peritumoral edema are features present in ~90% of gliomas [2,7,8,9,10,11,66,69,70,74,77,78,79,80]. However, intra-axial tumors are more likely to be associated with perilesional edema and mass effect than ischemic infarctions [8].

Particular MRI features that may assist with differentiation of canine astrocytomas from oligodendrogliomas or discriminating of low-grade from high-grade gliomas have been reported (Table 2). Compared with astrocytomas, canine oligodendrogliomas are more likely to be T1W hypointense, associated with smooth margins, contact the ventricles or brain surface, and distort the ventricles, and less likely to be associated with severe peritumoral edema [70,77,78]. Another study reported that the T2-FLAIR mismatch sign (Figure 5, Case 18), which is characterized by a lesion with a homogeneously hyperintense T2W signal and a hypointense signal with a hyperintense peripheral rim on FLAIR sequences, had 100% specificity for canine oligodendrogliomas, and more likely to be observed in low-grade tumors [66]. However, the predisposition for oligodendrogliomas for being T1W hypointense has been the only variable to emerge as significantly different between astrocytomas and oligodendrogliomas in more than one study [70,77]. Multiple studies have corroborated that contrast-enhancement and the presence of intratumoral cysts are more consistent (Table 2), but not universal, features among high-grade gliomas [69,70,77,78].

The heterogeneity of the MRI features of gliomas is reflected in the variability of reported diagnostic accuracies for the prediction of glioma, as well as glioma type (astrocytoma versus oligodendroglioma) and glioma grade. Analysis of qualitative MRI features to diagnose gliomas resulted in the correct identification of 1/3 (33%) of feline gliomas [9]. In the Snyder et al. [2], Rodenas et al. [10], and Thomas et al. [32] studies, MRI feature analysis resulted in the correct diagnosis of 6/9 (67%), 11/15 (73%) and 7/7 (100%) of canine gliomas, respectively. MRI features have been reported to be 84% sensitive and 94% specific for discrimination of gliomas from other brain lesions [11], 59–64% sensitive and 67–69% specific for identifying astrocytomas, 67% specific and 36% sensitive for predicting oligodendroglioma, and 60–67% sensitive and 40–57% specific for the identification of high-grade gliomas [70,74].

#### 3.3.2. Other Solitary Intra-Axial Tumors

**Figure 6:**
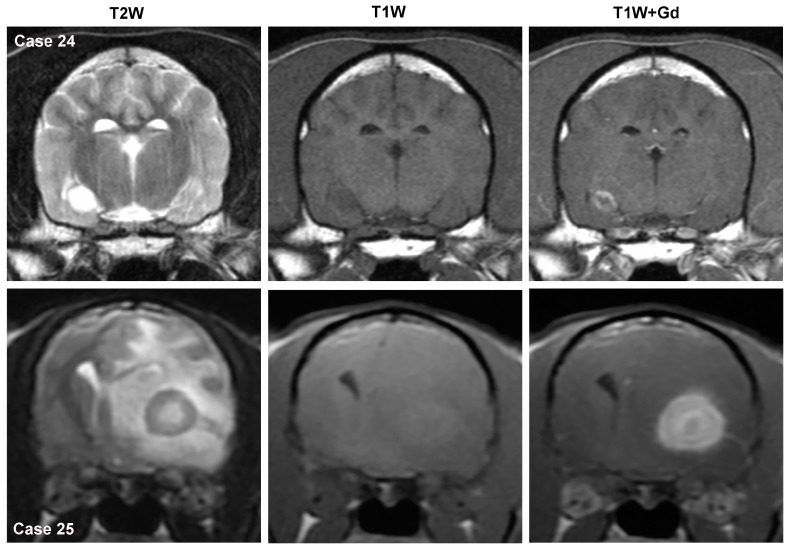
MRI features of solitary intra-axial brain metastases. Case 24—canine metastatic pulmonary carcinoma. The metastasis shares many features with glioma, including ovoid shape, T2W hyperintensity, T1W hypointensity, ring-type contrast enhancement, and location in the temporal lobe and hippocampus. Case 25—feline multicentric lymphoma with intracerebral metastasis. The tumor focus is heterogeneous on T2W images, T1W isointense, markedly contrast enhancing and associated with severe perilesional edema and mass effect.

These differential diagnoses include singular brain metastases, ependymoma, lymphoma, HS, and embryonal tumors [2,4,9,10,31,32,55,82,83]. The MRI features of most of these tumors appear elsewhere in this review. Solitary intra-axial brain metastases (carcinoma, hemangiosarcoma, melanoma, others) may have quite protean intrinsic MRI signal intensities, display variable sizes, severity, and patterns of contrast enhancement (Figure 6), and may or may not be associated with secondary brain changes as such as edema and mass effect [4,12,14,82]. As hematogenous metastasis disrupts the blood–brain barrier during extravasation to the brain, these lesions typically display some type of contrast enhancement [12]. Thus, these can be extremely difficult to discriminate from primary tumors or non-neoplastic inflammatory or vascular diseases based on MRI features alone, particularly when the existence of a distant primary tumor is unknown [7,10,11,12].

### 3.4. Solitary Intraventricular Mass Lesions

**Figure 7:**
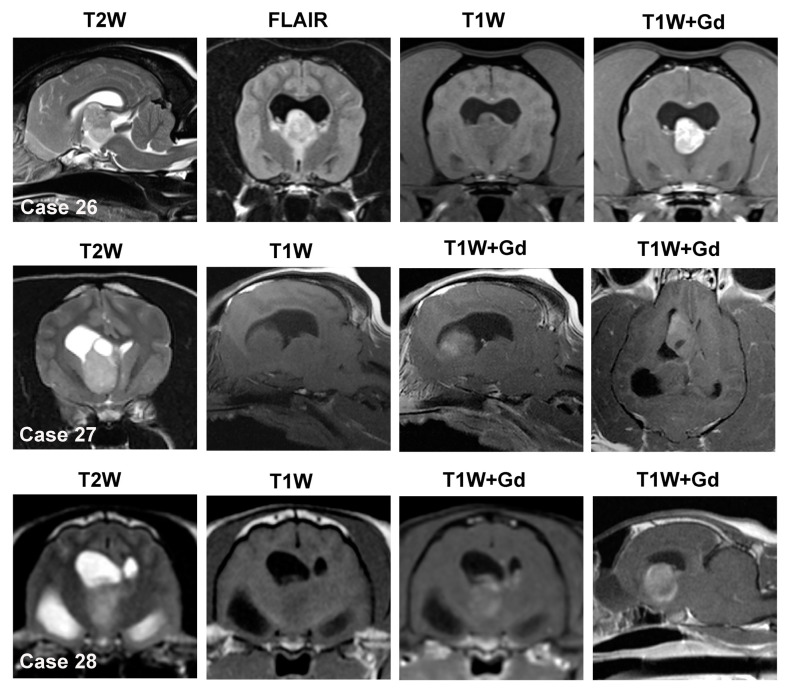
MRI features of solitary intraventricular masses. Case 26—canine choroid plexus papilloma involving the 3rd ventricle and interventricular foramina. The tumor is mixed T2W/FLAIR iso- to hyperintense, and T1W iso-to hypointense and demonstrates strong, uniform contrast enhancement. Case 27—canine intraventricular oligodendroglioma. The mass is T2W hyperintense, T1W isointense, contains a dorsal cystic region, and demonstrates scant partial contrast enhancement. Case 28—feline 3rd ventricular meningioma. The tumor is heterogeneous on T1W and T2W images and moderately and non-uniformly contrast enhancing. Each of these cases demonstrates evidence of obstructive hydrocephalus characterized by dilation of the lateral ventricle(s).

Intraventricular mass lesions encompass those found within the lateral ventricles, interventricular foramina, 3rd ventricle, mesencephalic aqueduct, fourth ventricle, or lateral apertures [12,14]. In the dog, CPTs (Figure 7, Case 26) are the most common primary intraventricular tumor, with intraventricular gliomas infrequent (Figure 7, Case 27) and ependymomas rare [1,2,31,70,81]. Nearly 50% of canine CPT are associated with the 4th ventricle, 22–36% with the 3rd ventricle, and 18–29% with the lateral ventricles [81]. In the largest MRI study of canine CPT, nearly two-thirds of the tumors were Grade III choroid plexus carcinomas (CPC), although prior neuropathologic studies reported Grade I choroid plexus papillomas (CPP) more commonly [2,31,81]. In the cat, meningiomas of the 3rd ventricle (Figure 7, Case 28) are the most common intraventricular tumor, followed by ependymoma, glioma, and CPT [3,73]. Lymphoma with intraventricular involvement may be seen in dogs and cats [57,58].

#### 3.4.1. Choroid Plexus Tumors

**Figure 8:**
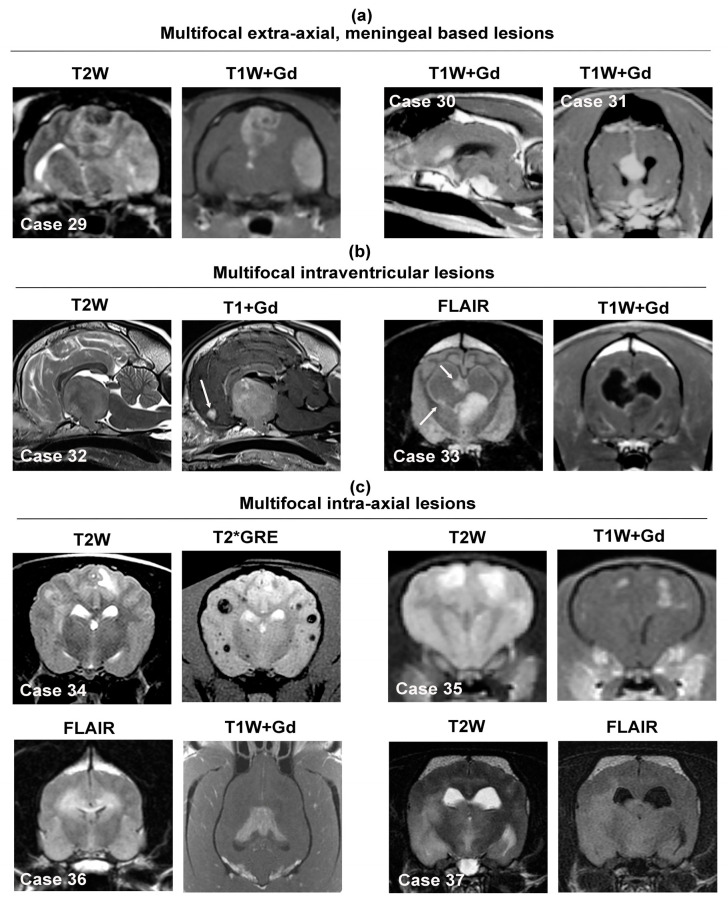
MRI features of multifocal intracranial neoplastic lesions. Multifocal extra-axial meningeal-based lesions appear in (**a**), multifocal intraventricular tumors in (**b**), and multiple intra-axial lesions in (**c**). Case 29—feline, multifocal parasagittal and cerebral convexity meningiomas. Case 30—canine multifocal extra-axial HS involving the falx cerebri and basilar region. Case 31—canine multifocal, uniformly contrast-enhancing falcine and parasellar meningiomas. Case 32—canine 3rd ventricular choroid plexus carcinoma with contrast-enhancing drop metastasis within the rostral horn of the lateral ventricle (arrow). Case 33—canine intraventricular high-grade oligodendroglioma with multiple ependymal drop metastases (arrows). Note the lack of suppression of the CSF signal on the FLAIR image. Case 34—canine multiple cerebral hemangiosarcoma metastases, which appear as heterogenous, round, variably sized T2W lesions that demonstrate susceptibility artifact on the T2*GRE sequence. Case 35—canine multifocal urothelial carcinoma metastases within the cerebrum; the metastatic foci or T2W hyperintense and demonstrate contrast enhancement. Case 36—canine ‘butterfly’ glioblastoma (high-grade astrocytoma) with bilaterally symmetric involvement of the subcortical white matter and corpus callosum. Case 37—canine gliomatosis cerebri manifesting as poorly defined T2W/FLAIR hyperintensities within the parietal, temporal, and piriform lobes and thalamus.

In the dog, an intraventricular mass is likely to be a CPT if it is effacing or replacing the choroid plexus architecture or if the normal choroid plexus cannot be identified on the MRI [14]. CPTs are iso- to hyperintense on both T1W and T2W sequences and typically demonstrate marked contrast enhancement (Figure 7) [31,81]. In the dog, the probability that an intraventricular mass is a CPT is also increased if the lesion is hyperintense on T1W images. CPP may display a papilliform shape, whereas this is rare in CPC [81]. Dilation of the ventricular system, which may occur rostral and/or caudal to the level of the intraventricular mass, obstructive hydrocephalus (Figure 7), peritumoral edema, intratumoral cysts, and hemorrhage are frequent features of CPT [31,81]. The diagnostic accuracies of MRI features for CPT range from 50–100%; however, in these reports, CPT was the only intraventricular tumor included [2,10,11,32]. CPT in the lateral apertures may have MRI characteristics that resemble cerebellopontine angle meningiomas [10]. CPT may also manifest as multiple intraventricular or subarachnoid contrast-enhancing masses (i.e., ‘drop-metastases’), which result from tumor dissemination through the cerebrospinal fluid pathways (Figure 8, Case 32). Drop metastases are most often associated with choroid plexus carcinomas. Intraventricular tumors may also occasionally be associated with inadequate suppression of cerebrospinal fluid signal (Figure 8, Case 33) on FLAIR sequences (i.e., intraventricular hyperintensity) due to severe CSF inflammation or increased protein content [73].

#### 3.4.2. Ependymoma

In the presence of an intraventricular mass, if a normal choroid plexus can be identified that is distinct from the other mass lesion, differential diagnoses should include tumors other than CPT, such as ependymoma or glioma. Ependymomas appear iso- to hyperintense on T1W images and T2W/FLAIR hyperintense [31,73] and are usually well-circumscribed, spherical masses in cats. In cats, a T1W hyperintense intraventricular mass is more likely to be an ependymoma than a CPT. Ependymomas may demonstrate a variety of contrast enhancement patterns, from none to subtle to marked [31,73]. Cystic structures, obstructive hydrocephalus, and peritumoral edema are also observed in ependymomas [73].

### 3.5. Multifocal Intracranial Neoplastic Mass Lesions

The major differential diagnostic considerations for multifocal intracranial neoplastic lesions are provided in Table 3. Several of the neoplasms that may present with multifocal mass lesions represent occasional to rarely observed variants of primary brain tumors that typically appear as solitary mass lesions (Figure 8, Cases 29–31) and were reviewed in Section 3.1, Section 3.2, Section 3.3 and Section 3.4. In these instances, other than the multifocal lesion distribution, the other imaging features and signal characteristics of these tumors are similar as to when they appear as solitary masses, and thus, only notable differences in lesion appearances or new tumor types from what have previously been covered are discussed in the sections below.

#### 3.5.1. Multifocal Intra-Axial Mass Lesions

This imaging presentation is predominantly caused by hematogenous metastases of systemic neoplasms such as hemangiosarcoma, carcinomas, or melanoma [4]. Intra-axial metastatic lesions are preferentially found in the telencephalon and typically with a distribution at gray-white matter interfaces. Metastatic hemangiosarcomas frequently present as poorly defined, heterogeneous lesions in all sequences, with many lesions containing susceptibility artifact on T2*GRE or SWI (Figure 8, Case 34), associated with moderate to severe perilesional edema, and demonstrate moderate to strong heterogeneous or ring-type contrast enhancement [12,14,82]. Metastatic carcinomas also appear as ill-defined lesions that may be T1W hypo- to iso-intense, T2W/FLAIR hyperintense, and are typically contrast enhancing, although the degree and patterns of contrast enhancement can vary significantly (Figure 8, Case 35). Unlike hemangiosarcoma, metastatic carcinoma lesions are not usually associated with imaging evidence of intratumoral hemorrhage. As melanin is a paramagnetic substance, melanomas may appear as hyperintense lesions on T1W sequences. Gliomas can manifest as bilaterally symmetric or asymmetric lesions with involvement of both cerebral hemispheres and the corpus callosum (i.e., butterfly glioma; Figure 8, Case 36), asymmetric multifocal to diffuse ill-defined parenchymal lesions (i.e., gliomatosis cerebri; Figure 8, Case 37), or multiple, discrete intra-axial masses (i.e., satellite lesions), all of which represent different manifestations of intraparenchymal glioma spread [70,71,72,75,76]. A notable characteristic of gliomatosis cerebri is its tendency to lack contrast enhancement [72,76].

#### 3.5.2. Multifocal or Diffuse Intraventricular Lesions

Multifocal intraventricular lesions most often result from drop metastases of choroid plexus carcinomas or gliomas with intraventricular involvement [70,71,81]. Choroid plexus carcinoma drop metastases are usually contrast-enhancing (Figure 8, Case 32), whereas glioma-associated drop metastases (Figure 8, Case 33) may have imaging characteristics that differ from the primary lesion, including a conspicuous lack of contrast enhancement of many lesions [71,81]. Lymphoma may also manifest with diffuse contrast-enhancing ependymal and periventricular lesion patterns [58,87].

#### 3.5.3. Multifocal Lesions Involving Multiple Neuroanatomic Locations

Lymphoma and HS are the tumors most likely to present with imaging evidence of disease simultaneously affecting multiple neuroanatomic locations [55,57,58,59]. Multifocal lesions may also be caused by the presence of multiple synchronous intracranial neoplasms of differing histologies. This situation has been described with various combinations of coexistent primary and secondary brain tumors (i.e., meningioma or glioma with lymphoma or pituitary tumor), multiple synchronous primary brain tumors (i.e., meningioma and glioma), and multiple secondary tumors (i.e., carcinoma and pituitary tumors) [2,3,4,9]. The presence of multiple lesions may also be due to separate diseases such as a meningioma or glioma with a contemporaneous cerebrovascular accident [43,71].

### 3.6. Quantitative MRI (qMRI) Methods and Applications in Veterinary Neuro-Oncology

Due to the imperfect sensitivities and specificities of quantitative MRI for brain tumor diagnosis and characterization of tumor biology, efforts have been made to investigate new non-invasive MRI techniques that can improve the overall diagnostic yield or the prognostic ability of neuroimaging. qMRI sequences encompass those that generate quantitative data that assist with the characterization of tissue properties, physiological functions, or metabolic processes [18,25,35]. Various qMRI techniques have been used to assist with the diagnosis of brain tumor type and grade, differentiate tumors from other intracranial lesions that can mimic neoplasms, and infer biological behavior (i.e., grade) of tumors.

#### 3.6.1. Diffusion-Weighted Imaging

The qualitative use of DWI/DTI (Figure 5) and its derived quantitative metrics, such as the ADC and FA, have been used to discriminate different types of brain tumors and were previously reviewed in the tumor-specific sections. Studies have evaluated the ability of DWI to differentiate brain tumors from inflammatory or vascular brain lesions in dogs [35,37]. Two investigations have reported that ADC and FA (Supplementary Table S1) values across several types of brain tumors, cerebrovascular diseases, and autoimmune meningoencephalitides demonstrate substantial overlap and are not significantly different enough to aid with the differentiation of these lesions [35,37]. One study reported a cutoff ADC value of ≥1.443 × 10^−3^ mm^2^/s was 100% specific as an indicator of brain neoplasia in dogs, but the sensitivity and accuracy of this cutoff value were low (35% and 56%, respectively) [35].

#### 3.6.2. Perfusion Weighted Imaging

**Figure 9:**
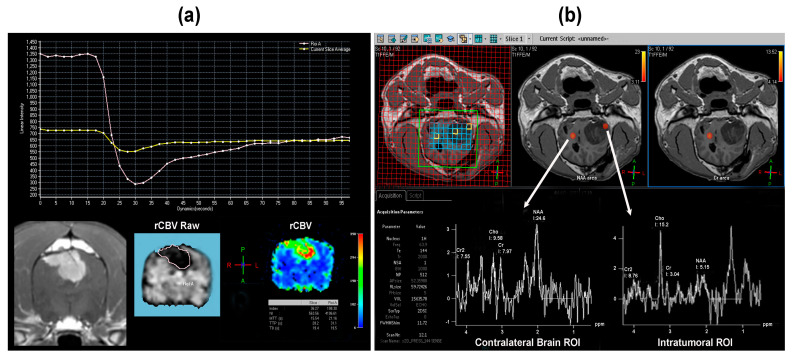
Applications of DCE-MRI (**a**) and ^1^H-MRS (**b**) studies in veterinary neuro-oncology. (**a**) DCE-MRI from a Grade II canine parasagittal meningioma was used to generate normalized relative cerebral blood volume (rCBV) of the tumor, with signal intensity curve, grayscale raw perfusion map (rCBV raw) with ROI, and graduated red-green-blue rainbow rCBV color map. The color scale (far right) depicts gadolinium signal intensity. The rCBV is significantly higher in the centrum of the tumor (red/yellow) than in the adjacent cerebral cortex (green) and underlying subcortical white matter (blue). (**b**) Multivoxel ^1^H-MRS spectra from canine high-grade oligodendroglioma using intratumoral as well as contralateral white matter of the brain ROI. Note the relative decrease in the NAA peak and increased choline (Cho) peak in the intratumoral ROI.

T1W-dynamic contrast enhancement perfusion MRI (DCE-MRI) analyzes the temporal enhancement pattern of the region of interest following the intravascular administration of a paramagnetic contrast agent. DCE-MRI is performed by acquiring baseline images prior to administration of the contrast agent, followed by longitudinal serial acquisition of images over a few minutes during and after the arrival of the contrast agent in the tissue of interest [18]. DCE-MRI allows for a non-invasive, quantitative evaluation of blood flow, tissue vascular density, integrity, and permeability. The insights derived from DCE-MRI findings (Figure 9a) can be utilized for investigating angiogenesis, tumor type and grade, hypoxia, evaluation of blood–brain barrier disruption (BBBD), and therapeutic effects in brain tumors [18,38]. There is little research in veterinary neuro-oncology regarding applications of DCE-MRI.

Zhao et al. performed DCE-MRI in seven dogs with four different types of primary and secondary brain tumors. They utilized a two-compartment pharmacokinetic model to calculate three enhancement parameters, E_R_ (rate of enhancement), K_el_ (rate of elimination), and K_ep_ (rate constant) and determined a model-free parameter initial area under the gadolinium curve (IAUGC) at 90 s. They found statistically significant differences between distributions of the enhancement patterns of each tumor type and moderate to strong correlations between the IAUGC model and the E_R_ parameter (r = 0.4–0.9), but the very limited sample size in this study precludes drawing conclusions about particular associations of these enhancement parameters with specific tumor types [38].

Subtraction enhancement analysis (SEA), which is another method to assess differences in gadolinium intensity signals within voxels acquired from T1W pre- and post-contrast sequences, can also be used to semiquantitatively evaluate neurovascular parameters such as BBBD. The advantage of SEA is that it is less technically and computationally demanding than DCE-MRI and can be readily performed on most open-source and proprietary image analysis software platforms. A two-armed study prospectively evaluated DCE-MRI and SEA and retrospectively the SEA of canine brain tumors [88]. Although the retrospective arm of the study included some histologically confirmed tumors, this was not a strict inclusion criterion, and no dogs in the prospective study arm had pathologically confirmed tumors, so this study was ultimately excluded from data extraction. However, it is summarized here as representing one of the few investigations of DCE-MRI for brain tumors [88].

To determine the SEA, the percentage of intensity difference (PID) was measured and compared to a temporal muscle reference [88]. The BBBD score was the percentage of positive voxels representing permeability and was determined in a (LR) low range (mean of PID in temporal muscle plus 1 SD) and a (HR) high range (mean PID in temporal muscle plus 2 SD). Better differentiation of tumor class (88% extra-axial and 90% intra-axial) was achieved using HR, and a significant correlation between tumor size and BBBD score was observed [88]. HT BBBD score alone did not differ significantly between the two tumor classes. LR-BBBD score and LR/HR ratio were significantly higher in gliomas than in meningiomas. A sensitivity of 80% and specificity of 100% were calculated for the ability of the SEA LR/HR ratio to distinguish between meningiomas and gliomas (AUC, 0.95 [CI, 0.87–1]) [88].

Other types of perfusion imaging methods, such as dynamic susceptibility contrast (DSC), arterial spin labeling (ASL), and blood oxygen level-dependent (BOLD) sequences, are available and are being used in veterinary clinical settings, but publications describing these techniques in dogs and cats with brain tumors were not identified [24].

#### 3.6.3. Proton Magnetic Resonance Spectroscopy (^1^H-MRS)

^1^H-MRS is a nuclear magnetic resonance technique that detects radiofrequency signals generated by spins of magnetic resonance active hydrogen nuclei (protons) precessing in an external magnetic field. As protons in different molecules resonate at slightly different frequencies due to local biochemical and magnetic microenvironmental conditions, this allows for the non-invasive, in vivo spatial detection and quantification of different metabolites containing protons based on characteristic chemical shifts in resonance frequency relative to water [36].

Few studies exist in veterinary medicine that evaluate ^1^H-MRS in brain tumors, with both reports investigating potential spectral differences between dogs with brain neoplasms and non-infectious meningoencephalitides [25,36]. Using a multivoxel ^1^H-MRS technique, Stadler et al. found that neoplastic lesions exhibited lower N-acetyl aspartate (NAA) and higher choline concentrations compared to inflammatory disease, resulting in a reduced NAA-to-choline ratio, decreased NAA-to-creatine ratio, and an elevated choline-to-creatine ratio [25]. Decreases in NAA are associated with neuronal destruction or damage, while elevations in choline signify increased cellular proliferation and membrane turnover, as would be expected from the presence of a tumor (Figure 9b). Significant differences in metabolites were not observed between the different tumor types included in the study [25]. Carrera et al. used single voxel ^1^H-MRS to demonstrate that dogs with brain tumors had significantly lower NAA, creatine, and glutamine-glutamate complex (Glx) concentrations and significantly higher choline concentrations than dogs with meningoencephalitis [36]. In this study, dogs with meningoencephalitis lacked histologic confirmation of the diagnosis [36].

#### 3.6.4. MRI-Based Radiomic Studies

‘Radiomics’ refers to the pixel/voxel-wise, high-throughput extraction of quantitative features from routinely acquired medical images (MRI, CT, PET, etc.) using computer software. A fundamental assumption of radiomics is that these features are biomarkers representative of certain characteristics of a disease process. These features can be shape-, texture-, or deformation-based [43]. Shape-based features include minor and major axes, elongation, shape, and sharpness. Shape-based features provide quantitative measures of tumor irregularity and boundary conditions based on their three-dimensional topology. Texture-based features, such as smoothness, kurtosis, entropy, contrast/intensity, mean, and homogeneity, investigate pixel-level differences within the lesion to characterize how heterogeneous a tumor is. Deformation-based features capture the impact of the lesion relative to the surrounding tissue, such as the mass effect. Using mathematical algorithms or codes, computer software can learn from the feature data to recognize patterns that are not easily discerned by the human eye and select the features that perform the best to predict the desired task [43,44,45].

There are several studies describing MRI-based radiomics in veterinary neuro- oncology [34,43,44,45,67]. One study reported the use of texture features to differentiate between canine benign and atypical/anaplastic meningiomas with 96.8% sensitivity and 92% specificity [43]. Another study employed two deep learning models, a transfer-learning neural network (Deep Neural Networks; DNN) with previously trained images and another DNN developed from ‘scratch’ for the prediction of canine meningioma grade [45]. The pre-trained DNN generated 65–68% prediction accuracies, while the scratch DNN resulted in accuracies ranging from 75–82% [45]. The same research group also investigated the use of a pre-trained DNN to differentiate between canine meningiomas and gliomas, reporting accuracies of 94% using features derived from post-contrast T1W images, 91% using pre-contrast T1W images, and 90% using T2W images [44].

Wanamaker et al. used texture-based radiomics to assess for differences between neoplastic and inflammatory brain diseases in dogs and to predict different types of glioma and histopathological variants of autoimmune meningoencephalitides. This study reported a classification accuracy of 85% (89% sensitivity and 81% specificity) for glioma and meningoencephalitis. However, the classification accuracies of texture-based features to identify the type and grade of gliomas and the specific subtypes of autoimmune meningoencephalitides were all too low to be clinically useful [34]. Barge et al. extracted texture-based features from 40 canine gliomas, analyzed those features using three machine learning classifiers, and built models to predict tumor type (astrocytoma vs. oligodendroglioma) and grade (high vs. low) [68]. The average reported accuracies for the machine learning classifiers were 77% for discriminating tumor types and 76% for predicting high-grade gliomas. Across the machine learning methods, the support vector machine classifier demonstrated superior performance, with accuracies of 94% for predicting tumor types and 87% for tumor grade [68]. Texture features extracted from peritumoral edema in T1W images and from non-enhancing tumors in T2W were demonstrated to be more discriminative for the tumor type and grade, respectively [68].

## 4. Discussion

This review provides a contemporary summary of the qualitative and quantitative MRI features of canine and feline brain tumors, as well as an MRI feature-based conceptual framework to facilitate the generation of neuroimaging differential diagnoses in clinical practice. To optimize the diagnostic yield of brain MRI, imaging data should be interpreted alongside all available clinicopathologic information. The published literature indicates MRI acquisition protocols should minimally consist of multiplanar (or 3D) T2W images, multiplanar (or 3D) T1W images pre- and post-contrast, FLAIR, T2*GRE or SWI, and DWI/DTI sequences [6,7,8,11,12,13,14,24]. A holistic approach to MRI interpretation that incorporates lesion number, location within the brain, shape, intrinsic signal appearances on multiparametric sequences, patterns of contrast enhancement, and associated secondary changes in the brain can facilitate prioritization of differential diagnoses, reasonably discriminate neoplastic from non-neoplastic etiologies of brain disease, and often allows for accurate presumptive diagnosis of common types intracranial tumors in both dogs and cats, such as meningiomas, gliomas, CPT, and pituitary tumors [1,7,8,9,10,11,12,13,14]. However, there are numerous distinct neuropathologic primary and secondary brain tumor entities for which there exist limited or no MRI descriptions, particularly in cats, and definitive diagnosis of a brain tumor requires histopathologic examination of representative tissue [1,12,13,14].

Although various iterations of the algorithmic approach presented here have been utilized to streamline the imaging diagnosis of brain tumors, challenges remain at nearly all stages of clinical application of the algorithm [7,10,12,13,14,31,32]. For example, when attempting to classify the neuroanatomic location of the lesion, it can be difficult to distinguish a peripherally located intra-axial lesion from an extra-axial lesion or an intra-axial lesion invading or distorting the ventricle from an intraventricular tumor [7,10,67,70]. The substantial degree of overlap of lesion morphologies, intrinsic signal characteristics, and contrast-enhancement patterns that exist between the various tumor types that can be found in each neuroanatomic location diminishes the specificity of these imaging features for any particular tumor type [7,11,12,13,14,77,78]. The preponderance of evidence indicates that, in the absence of a lesion demonstrating T2-FLAIR mismatch (which is only present in a minority of oligodendrogliomas), reliable prediction of glioma type and grade, nor accurate classification of meningioma or pituitary tumor grades are not currently possible in dogs using qualitative MRI feature assessments [48,65,66,71]. Further, while some imaging features, such as the presence of a T2-FLAIR mismatch sign, claw sign, drop-metastases, and vessel susceptibility sign, can facilitate the identification of oligodendrogliomas, intra-axial lesions, choroid plexus carcinomas, and IVL, respectively, qualitative MRI features that have been robustly substantiated by multiple studies to be pathognomonic for neoplasia in general, or any tumor type or grade have yet to be identified [60,65,66].

Statistical comparisons of the extracted data were not attempted in this review due to the limitations and biases associated with retrospective cohort studies and case series, which comprised the vast majority of source data. Small sample sizes were common. Among the 59 studies reporting canine MRI data in this review, 66% (39/59) included tumor groups represented by ≤30 samples [2,6,8,25,30,32,33,37,38,39,40,43,47,50,51,54,55,56,58,59,60,67,71,72,74,75,76,78,79,80,82,83], with only 2/59 (3%) containing tumor sample sizes >100 cases [48,66]. The feline data further exemplifies the breadth of this issue. Over the 28-year study period, a total of 125 pathologically confirmed feline brain tumor cases with correlative MRI studies were identified, and 54/125 (43%) of these cases described tumors other than meningiomas [9,26,41,49,51,57,58,59,73]. While these data are not an exhaustive summary of the published literature as our inclusion criteria did not consider studies with ≤3 cases, they illustrate the data void that exists for brain tumors. Sample size bias effects may be further compounded in those studies with small or imbalanced analytical subgroups. This situation was observed repeatedly in canine studies attempting to identify differences among tumor grades (i.e., high-grade vs. low-grade gliomas or Grade I vs. Grade III meningiomas), owing to the lower prevalence of low-grade gliomas and rarity of malignant meningiomas in dogs and cats [34,42,44,46,66,68,69,70].

The subjectivity and observer bias inherent to qualitative MRI assessments further complicates uniform cross-study comparisons [6,7,8,9,10,11,66,77]. While there are common conventions and perceptions of what narrative descriptions of certain imaging features mean, study-specific definitions of or imaging inclusion criteria for these index test features are frequently not provided or standardized, which can add further ambiguity to interpretation. For example, what specific imaging criteria must be present in a ‘dural tail’ sign [9,12,14,40,51]? What constitutes a heterogeneous signal intensity? Is there a threshold that distinguishes lesion contact with the ventricle from distortion of the ventricle [70,77,78]? Does an intratumoral cyst differ in size or signal characteristics from intratumoral fluid [47,48]? Should semiquantitative gradations of contrast enhancement be derived from the volume of enhancing tissue or the relative signal intensity of the enhancement? There also exists the possibility for lesion misclassification errors in multiple studies, particularly in relation to tumor typing and grading, as interobserver variability, even among experienced neuropathologists, is a well-recognized phenomenon [66,69,70,89]. These factors could contribute to the inability to identify specific and significant imaging variables associated with tumor type or grade across different studies.

There are significant knowledge gaps with respect to MRI characteristics of tumors that have prognostic value. In canine gliomas, MRI features such as poorly or irregularly marginated tumors, the presence of drop metastases, and tumor T2W heterogeneity have been identified as risk factors for shorter survival [70,71]. However, these imaging characteristics have not been evaluated in large populations of dogs that received uniform treatment. Pituitary tumor size and involvement of local neurovascular structures have been shown to adversely influence the likelihood of complete tumor excision via transsphenoidal hypophysectomy [63,64]. Other studies included in this review did not identify prognostic MRI tumor features. This may be at least partially due to the current lack of data definitively associating tumor neuropathological features, such as tumor grade, with prognosis, as well as limited veterinary studies describing both MRI tumor characteristics and therapeutic outcome data [1,70].

On the population level, qMRI techniques coupled with machine learning methods show promise for the non-invasive discrimination of neoplastic from non-neoplastic brain lesions and to provide insight into various pathobiological features of brain tumors [34,44,45,46,68]. The current data indicate that radiomic classification accuracies for veterinary brain tumors are not superior to those obtained by qualitative assessments by experienced reviewers [34,44,45,46,68]. This has practical cost:benefit implications, as radiomic analytic workflows are time-consuming and require the use of multiple software platforms. In addition, given the wide range of qMRI value distributions across disease categories and the overlap between tumor types, qMRI variables have limited utility for the diagnosis of disease in individual cases. The radiomic studies in this review also share the small sample size limitations that have been previously discussed, which predisposes to overfitting of computational models [28]. Substantial methodological variation with respect to image processing and analytical techniques also prohibited comparisons between radiomic studies. Additionally, algorithmic bias (data leakage) could be a factor in those studies in which radiomic feature selection was performed before cross-validation [28,68]. In veterinary medicine, there is a need for critical appraisal and standardization of the many imaging pre-processing steps and analytical methods required to conduct radiomic studies [28]. Given the pervasive sample size limitations in veterinary neuro-oncology, it is also highly likely that rigorously designed and robustly powered qMRI studies will require multi-institutional collaborations.

Centrally organized imaging and clinical data registries could provide opportunities to improve the quality, scope, and power of MRI studies of canine and feline brain tumors. Such infrastructural resources already exist that could facilitate the conduct of multi-institutional brain tumor imaging-based research, and there are precedents of their use in veterinary neuro-oncology [90]. The National Cancer Institute’s Cancer Imaging Program has a publicly accessible, cloud-based service called the Cancer Imaging Archive (TCIA) that contains fully anonymized and neuropathologically annotated diagnostic imaging studies from humans and dogs with brain cancers, as well as an evolving suite of freeware tools to assist with analysis of the hosted data [90].

## 5. Conclusions

MRI examination of the brain is an indispensable and versatile diagnostic tool for the evaluation of dogs and cats with clinical signs of intracranial disease. Qualitative MRI feature analysis often allows for the differentiation of neoplastic from other etiologies of brain disease, as well as an accurate prediction of common types of brain tumors affecting dogs and cats [7,11]. However, there are several brain tumors that share similar MRI features with non-neoplastic diseases, as well as other types of brain tumors that can make presumptive diagnoses based on imaging features challenging. There are also many uncommon to rare brain tumor types for which the MRI features have not been described in detail, especially in cats. Robustly powered qualitative and quantitative MRI studies in veterinary medicine will likely require multi-institutional collaborations in order to harmonize image processing and analysis, as well as include sufficient numbers of ‘gold-standard’ cases with histopathologically confirmed, consensus diagnoses.

## Figures and Tables

**Table 1:** MRI features a summary of meningiomas and canine extra-axial histiocytic sarcoma.

MRI Feature	Feline Meningioma [9,26,41,49] (Proportion) *	Canine Meningioma [2,7,10,26,30,31,32,33,39,40,47,48,50,51,52,53,54] (Proportion) *	Canine Histiocytic Sarcoma [2,33,52,53,54,55,56] (Proportion) *
Predominant T1W Signal Intensity			
Isointense	42%	33% (12–82%)	25% (17–100%)
Hypointense	69% (58–100%)	33% (12–88%)	83% (75–100%)
Hyperintense	NR	19% (16–20%)	NR
Predominant T2W Signal Intensity			
Isointense	20% (9–33%)	24% (12–44%)	40% (14–50%)
Hypointense	3%	8% (2–14%)	33% (29–50%)
Hyperintense	80% (76–88%)	78% (44–100%)	24% (17–47%)
Predominant PDW Signal Intensity			
Isointense	6%	40% (12–68%)	71%
Hypointense	6%	88%	NR
Hyperintense	88%	66% (32–100%)	NR
Contrast Enhancement Present	100%	100% (94–100%)	100% (present in all cases)
Contrast Enhancement Uniformity, Severity, Pattern			
Homogeneous	~50%	75% (39–78%)	52% (43–60%)
Heterogeneous	~50%	61% (34–88%)	39% (29–57%)
Marked	91%	86% (78–100%)	69% (67–71%)
Mild-moderate	9%	17% (12–22%)	31% (22–33%)
Ring-like ^a^	12%	11% (2–22%)	14% (10–18%)
Calvarial Hyperostosis Present	73%	25% (23–27%)	12%
Cystic Regions Present	6%	22% (13–32%)	15% (12–17%)
Dural Tail Present	64%	25% (22–91%)	53% (29–100%)
Mass Effect Present	97%	96% (75–100%)	100% (present in all cases)
Peritumoral Edema Present	79% Mild in 58%	89% (60–100%) Severe in 40–50%	100% (92–100%) Severe in 60–72%

NR = not reported. * When data extracted from multiple studies, median and ranges provided. ^a^ This category considered subset of mild/moderate heterogenous enhancement.

**Table 2:** MRI features significantly associated with canine glioma type or grade in at least one study.

MRI Feature and Study [Ref]	Tumor Type		*p*-Value *
	Oligodendroglioma Proportion	Astrocytoma Proportion	
Severe Peritumoral Edema			
Bentley, et al., 2013 [77] ^a^	21%	40%	0.0003
Jose-Lopez et al., 2021 [70] ^b^	20%	47%	0.28
Smooth/Regular Margins			
Young, et al., 2011 [78]	81%	79%	NR
Bentley, et al., 2013 [77]	57%	55%	0.82
Jose-Lopez et al., 2021 [70]	69%	47%	0.02
Surface Contact			
Young, et al., 2011 [78]	94%	50%	0.046
Jose-Lopez et al., 2021 [70]	77%	78%	0.65
Ventricular Contact			
Young, et al., 2011 [78]	94%	93%	1.0
Jose-Lopez et al., 2021 [70]	69%	47%	0.05
Ventricular Distortion			
Bentley, et al., 2013 [77]	84%	66%	0.006
Jose-Lopez et al., 2021 [70]	88%	92%	0.82
T1W Hypointensity			
Young, et al., 2011 [78]	86%	75%	0.25
Bentley, et al., 2013 [77]	76%	93%	0.006
Jose-Lopez et al., 2021 [70]	96%	47%	<0.001
T2-FLAIR Mismatch [66]	16%	0%	<0.0001
	Tumor Grade		
	High-Grade ^c^	Low-Grade	
Contrast Enhancement Present ^d^			
Young, et al., 2011 [78]	87%	20%	0.008
Bentley, et al., 2013 [77]	78%	53%	0.0004
Jose-Lopez et al., 2021 [70]	76%	50%	0.37
Amphimaque, et al., 2022 [69] ^e^	100%	63%	0.002
Intratumoral Cysts/Fluid ^f^			
Young, et al., 2011 [78]	68%	40%	0.327
Bentley, et al., 2013 [77]	70%	31%	<0.04
Jose-Lopez et al., 2021 [70]	40%	43%	0.99
Amphimaque, et al., 2022 [69] ^e^	79%	38%	0.042
Invasion of adjacent brain			
Jose-Lopez et al., 2021 [70]	75%	29%	0.02
T2-FLAIR Mismatch [66] ^e^	33%	4%	0.001

NR = not reported. * *p*-values extracted directly from data sources, with all studies reporting significance at *p* < 0.05. ^a^ Qualitatively reported as combined moderate/severe edema. ^b^ Quantitative severe edema threshold reported. ^c^ Includes Grade III and/or Grade IV tumors. ^d^ Includes any contrast-enhancement pattern or severity. ^e^ Sample population exclusively oligodendrogliomas. ^f^ Includes single or multifocal cystic lesions.

**Table 3:** MRI differential diagnoses for multifocal or diffuse intracranial neoplastic lesions.

Extra-Axial, Meningeal Based Masses	Sellar/ Parasellar Masses	Intra-Axial Masses	Intraventricular Masses	Multiple Locations
Meningioma	Lymphoma	Brain metastases	Choroid plexus tumor with drop metastases	Lymphoma
Lymphoma	Histiocytic sarcoma	Lymphoma	Glioma with drop metastases	Histiocytic sarcoma
Histiocytic sarcoma	Meningioma	Histiocytic sarcoma	Lymphoma	Meningioma
Brain Metastases	Brain Metastases	Glioma		
Distinct, synchronous tumors	Distinct, synchronous tumors	Distinct, synchronous tumors	Distinct, synchronous tumors	Distinct, synchronous tumors

## Data Availability

Data are contained within the article.

## Supplementary Materials

The following supporting information can be downloaded at: https://www.mdpi.com/article/10.3390/ani14071044/s1; Table S1: Apparent diffusion coefficient (ADC) and fractional anisotropy (FA) values for different intracranial lesions in dogs and cats.
