# Supplementary material for: An Illustrated Scoping Review of the Magnetic Resonance Imaging Characteristics of Canine and Feline Brain Tumors
Source: Animals (Basel). 2024 Mar 29;14(7):1044. doi: 10.3390/ani14071044 (PMC11010916; doi:10.3390/ani14071044)
Supplement: Supplementary file 1 [file animals-14-01044-s001.zip › animals-2908667-supplementary.pdf]

Supplemental Table S1- Apparent diffusion coefficient (ADC) and fractional anisotropy (FA) values for different intracranial lesions in dogs and cats

**Lesion Type**[Reference]	**ADC Dogs (10^{-3} mm^2/s)**	**ADC Cats (10^{-3} mm^2/s)**	**FA Dogs (10^{-3} mm^2/s)**	**FA Cats (10^{-3} mm^2/s)**
**Non-neoplastic Lesions**				
Non-infectious meningoencephalitis[35] (median, range)	0.88 (0.5-1.36)	NR	0.17 (0.13-0.59)	NR
Non-infectious meningoencephalitis[37] (mean [SD])	1.14 [0.22]	NR	NR	NR
Acute ischemic infarction[37] (mean [SD])	0.68 [0.25]	NR	NR	NR
Chronic ischemic infarction[37] (mean [SD])	1.1 [0.24]	NR	NR	NR
Hemorrhagic infarction[37] (mean [SD])	1.64 [0.44]	NR	NR	NR
**Neoplastic Lesions**				
Meningioma				
• Intratumoral ROI (Small)[26] (median, range)	0.98 (0.54-1.45)	0.72 (0.64-0.78)	0.19 (0.1-0.3)	0.24 (0.20-0.33)
• Peritumoral ROI[26] (median, range)	0.95 (0.51-1.16)	0.83 (0.76-0.97)	0.3 (0.14-0.44)	0.38 (0.31-0.42)
• Intratumoral ROI[35] (median, range)	0.93 (0.56-3.29)	NR	0.28 (0.08-0.42)	NR
• Intratumoral ROI[37] (mean [SD])	1.32 [0.34]	NR	NR	NR
• Intratumoral ROI (Small)[54] (median, range)	1.07 (0.8-1.21)	NR	0.18 (0.13-0.23)	NR
• Peritumoral ROI[54] (median, range)	0.93 (0.82-1.18)	NR	0.29 (0.13-0.44)	NR
• Grade I (Mean)[42] (mean [SD])	1.15 [0.16]	NR	NR	NR
• Grade II (Mean)[42] (mean [SD])	1.0 [0.19]	NR	NR	NR
• Grade III (Mean)[42] (mean [SD])	1.23 [0.22]	NR	NR	NR
Histiocytic Sarcoma				
• Intratumoral ROI (Small)[54] (median, range)	0.76 (0.70-0.93)	NR	0.16 (0.12-0.27)	NR
• Peritumoral ROI[54] (median, range)	1.08 (0.94-1.2)	NR	0.24 (0.19-0.32)	NR
Pituitary tumors[37] (mean [SD])	0.91 [0.25]	NR	NR	NR
Glioma[35] (median, range)	0.88 (0.38-2.5)	NR	0.18 (0.07-0.56)	NR
Glioma[37] (mean [SD])	0.96 [0.36]	NR	NR	NR
Choroid plexus tumor[37] (mean [SD])	1.64 [0.35]	NR	NR	NR
Metastatic hemangiosarcoma[35] (median, range)	0.95 (0.18-1.48)	NR	0.3 (02.4-0.67)	NR

NR= not reported
ROI= region of interest
